# Complex Tibial Plateau Fractures: Primary Fixation Using the Ilizarov External Fixator. A Two-year Study at Civil Hospital Karachi, Pakistan

**DOI:** 10.7759/cureus.5375

**Published:** 2019-08-13

**Authors:** Ahmed Raza, Sunil Kumar, Dileep Kumar, Abdul Qadir, Muhammad Muzzammil, Mohammad Tahir Lakho

**Affiliations:** 1 Trauma and Orthopedic Surgery, Dow University of Health Sciences, Karachi, PAK; 2 Trauma and Orthopedic Surgery, Civil Hospital Karachi, Dow University of Health Sciences, Karachi, PAK; 3 Orthopedic Surgery, Dr. Ruth KM Pfau Civil Hospital Karachi, Dow University of Health Sciences, Karachi, PAK; 4 Orthopedic Surgery, Civil Hospital Karachi, Dow University of Health Sciences, Karachi, PAK; 5 Orthopedics, Dr. Ruth KM Pfau Civil Hospital Karachi, Karachi, PAK

**Keywords:** complex intra-articular, ilizarov external fixator, tibial plateau fractures

## Abstract

Background

The proximal tibia with the meta-diaphysis junction is a critical weight-bearing area. An injury around this region may be restricted to the tibia or associated with a significant soft-tissue injury. The objective of the present study is to assess the results of closed reduction and Ilizarov external fixation in the management of complex tibial plateau fractures.

Patients and methods

The study included 26 patients with high-energy tibial plateau fractures (Schatzker types V and VI). The ages ranged from 23 to 60 years, with an average of 35 years. The trauma was a road traffic accident in 19 cases and a fall from a height in eight cases. The fractures were closed in 18 cases and open in five. The open fractures were Gustilo-Anderson type I in three cases and type II in five cases. Soft-tissue injuries associated with closed fractures were classified according to the Tscherne system. The follow-up period averaged 24 months. The average time of surgery was 85 mins (range: 60-120 min). The mean time to union was 12 weeks. At the final follow-up, the average total range of knee flexion was 120° (range: 0-170°).

Results

Results were satisfactory in 22 cases and unsatisfactory in four cases according to Rasmussen's knee functional score. Complications included pin-tract infection in 10 cases, an extension lag in three cases and varus deformity of about 17° in one case.

Conclusion

Hybrid external fixation is a good method for the treatment of comminuted tibial plateau fractures. It allows for early joint movement and reduces the risk of serious complications.

## Introduction

Bicondylar tibial plateau fractures are often associated with severe soft-tissue injuries that can affect the results of treatment. The operative management of these injuries is invariably complicated by the condition of the soft-tissue envelope of the proximal tibia [1].

A biological approach to the soft-tissue envelope of the proximal tibia can help minimize some of the complications that often follow surgical intervention [2].

The aim of this study is to evaluate the hypothesis of whether the minimally invasive technique of Ilizarov external fixator with minimal internal fixation in tibial plateau fractures can provide a satisfactory outcome with few complications. Particular attention is paid to the functional outcome of, and the complications associated with, this treatment method.

## Materials and methods

Between January 2017 and March 2018, 40 patients with high-energy bicondylar tibial plateau fractures were managed with the use of an Ilizarov circular external fixator. There were 33 men and seven women. The mean age was 30 years (range: 23-56 years). Exclusion criteria were patients with concomitant injuries that could alter the functional outcome of the patient, for example, an ipsilateral femoral shaft fracture, an ipsilateral acetabulum fracture, and bilateral fractures. Six patients met these criteria.

**Figure 1:**
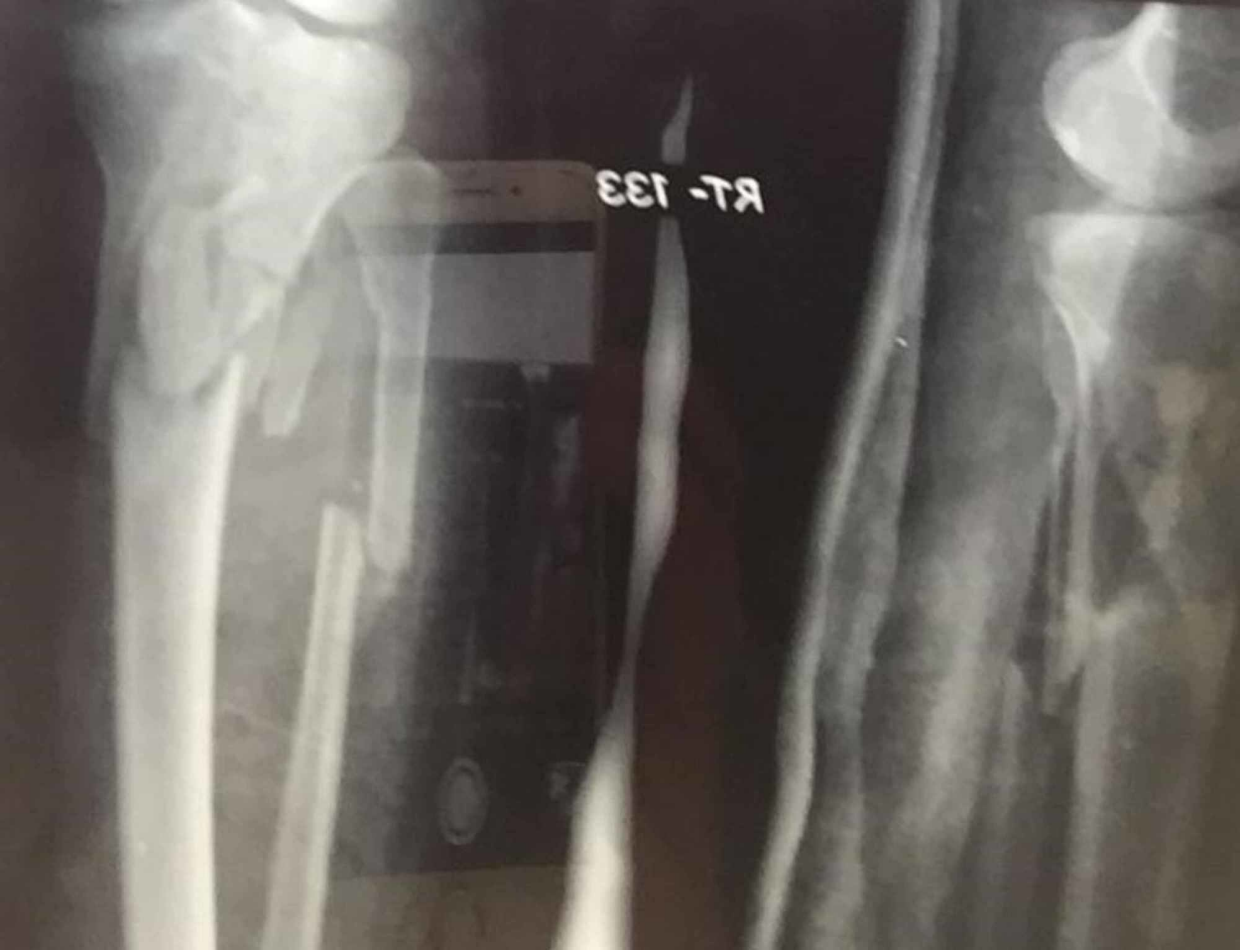
Preoperative plain radiograph of one patient showing a comminuted tibial plateau fracture, Shatker's type VI, along with a fracture of the proximal part of the fibula

The mechanism of injury was a road traffic accident in 11 cases and a fall from a height in nine cases. The fractures were closed in 16 cases and open in four cases. The open fractures were Gustilo-Anderson type I in one case and type II in three cases. Soft-tissue injuries associated with closed fractures were classified according to the Tscherne system [3]. Eleven fractures were Schatzker type V and nine cases were type VI [4]. Computed tomography was performed in all cases, to assess the degree of comminution and the amount of depression and to detect the main fragments through which the screws could be inserted (Figure 1).

Prophylactic antibiotics were administered intravenously in all cases. In the open fracture cases, antibiotics were prescribed as necessary for the first days and subsequently replaced according to the culture results. All open fractures initially received a combination of a second-generation cephalosporin with an aminoglycoside.

The procedures were performed under spinal anesthesia. The patients were positioned supine on a radiolucent fracture table. Reduction was achieved with traction given by the assistant, and manipulation was done by the surgeon. The fracture reduction was visualized with an image intensifier. Through a small incision over the anteromedial aspect of the tibial metaphysis, a small "window" was made in the tibial cortex in nine cases. A blunt-tipped, curved 3-mm K-wire or a simple pusher was inserted through the hole, up to the articular fragments, which were elevated under the image intensifier control. Bone grafts were applied to feel the osseous gaps in three cases. After achieving adequate reduction, three K-wires (1.8-mm thick) were placed in the juxta-articular bone parallel to the joint line. Each wire was positioned centrally in the major fracture fragment and perpendicular to the major fracture lines so as to hold the reduction. These wires are tensioned over an appropriate-sized ring. A preassembled tibial frame consisted of two appropriately sized rings, and the distal ring was placed 2-3 cm proximal and parallel to the ankle joint. The proximal ring of this frame was placed 2-3 cm distally to the fracture. Three tensioned wires were attached to each ring. Finally, the distal preassembled frame is connected to the proximal ring using four threaded rods to reduce the diaphysis portion of the fracture to the metaphyseal one. Additional fixation was done with percutaneously placed 4 or 6.5 mm cannulated cancellous screws to achieve intercondylar compression in nine patients. A femoral frame was applied in three cases for marked fracture comminution, necessitating more proximal stability over the knee.

Postoperative care

Exercise of the isometric quadriceps was started from postoperative Day 1. Early knee motion was encouraged from the first week. Touch-down weight-bearing was allowed initially and then advanced to partial weight-bearing as tolerated as possible at three weeks. Full weight-bearing was allowed when a complete union was achieved within four months. Serial radiographs were taken at four-week intervals to detect any deviation of mechanical axis and union, which was defined as an obliteration of the major fracture lines in both views.

The pin site was cared for daily with alcohol. After the bone had healed, frame dynamization was done. The external fixator was removed under anesthesia. After removal of the external fixator, clinical and radiological evaluations were made every three months for the first year and then every six months until the final follow-up. The results were evaluated according to the Rasmussen knee functional score [5]. The patients’ satisfaction was evaluated through an interview in which they subjectively reported their level of satisfaction (from full satisfaction to dissatisfaction). The patients’ level of satisfaction was assessed by asking them how they felt after the surgical procedure compared with how they felt before the operation [6]; whether they would go through the procedure again [7]; and whether they would recommend it to other people who sustained a similar fracture [8].

## Results

The follow-up period averaged 31 months (range: 18-45 months). There was no incidence of nonunion, septic arthritis or deep-vein thrombosis. The mean time of union was 3.2 months (range: 2.5-3.5 months). According to Rasmussen's knee functional score [9], the results were excellent in five cases, good in 12 cases, fair in two cases, and poor in one case. The mean duration of surgery was 100 min (range: 80-150 min). The mean trauma to surgery interval was 23 h (range: 5-74 h). The average hospital stay was 11 days (Figure 1).

**Figure 2:**
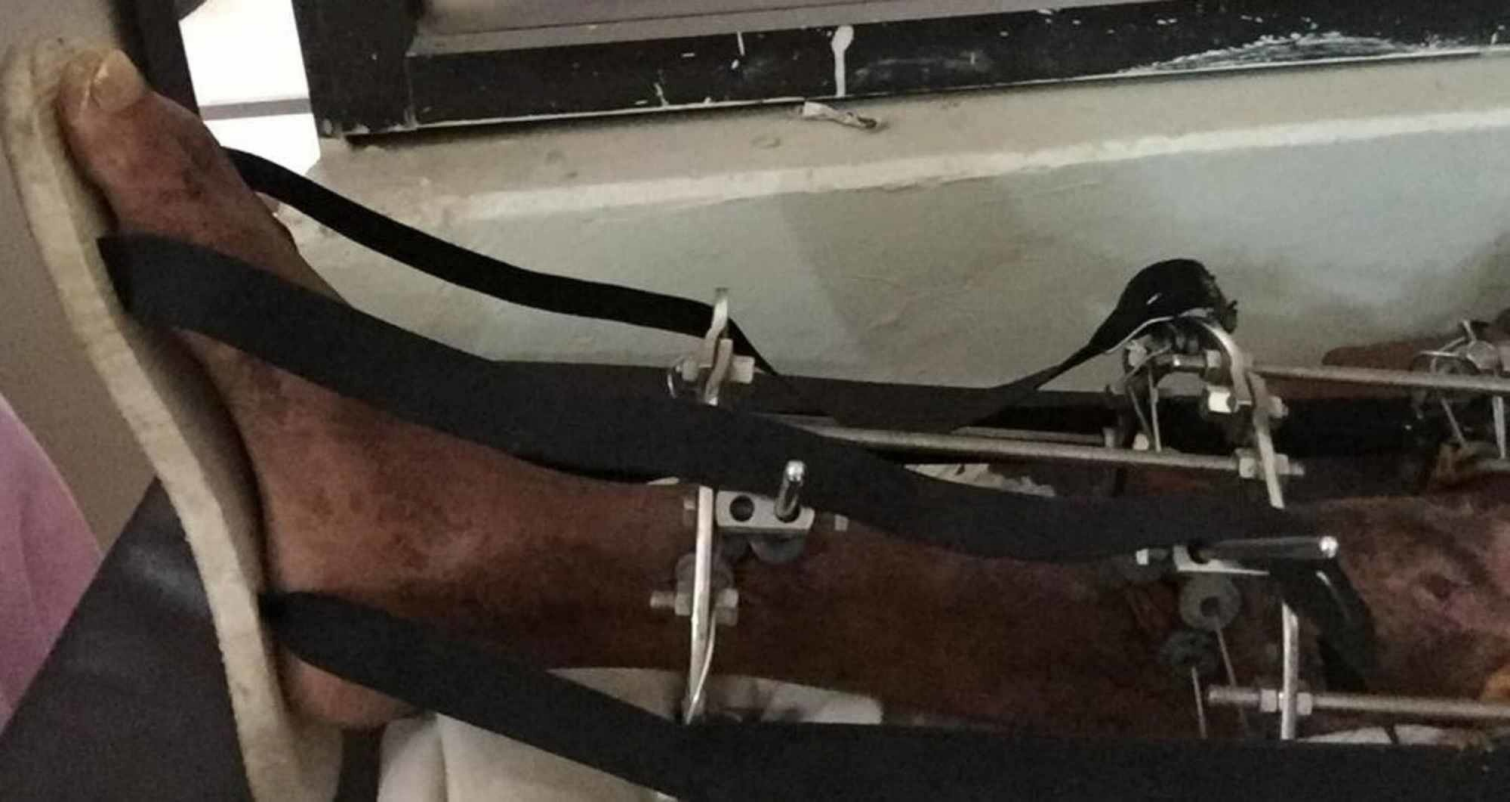
Postoperative picture of the Ilizarov fixator with the lower limb in alignment

The external fixators were removed on an average of 3.5 months (range: 2.8-5 months) without additional immobilization. Skin graft coverage was needed only for one patient. The average total range of knee flexion was 112.5° (range: 0-170°), but three patients had a total arc of motion of less than 60° and two of them had a femoral extension of the fixator. All patients received physiotherapy after removal of the frame. Extension lag was a common finding. It was observed in five cases; however, they showed gradual improvement of range of motion that was compatible with a normal gait within six months. One patient had a positive anterior drawer test but without functional instability. None of the cases had mediolateral instability. At final follow-up, radiographs showed articular depression more than 3 mm in three (15%) cases and less than 3 mm in four (20%) cases. The quality of reduction increased the functional score. The patients’ satisfaction was significantly related to functional results (p < 0.05). In 20% of the cases, pin-site infections were observed. These infections were superficial or limited to the soft tissue (Figure 2). This was controlled by frequent dressing and local antibiotics. No loss of reduction occurred after removal of the fixator.

**Figure 3:**
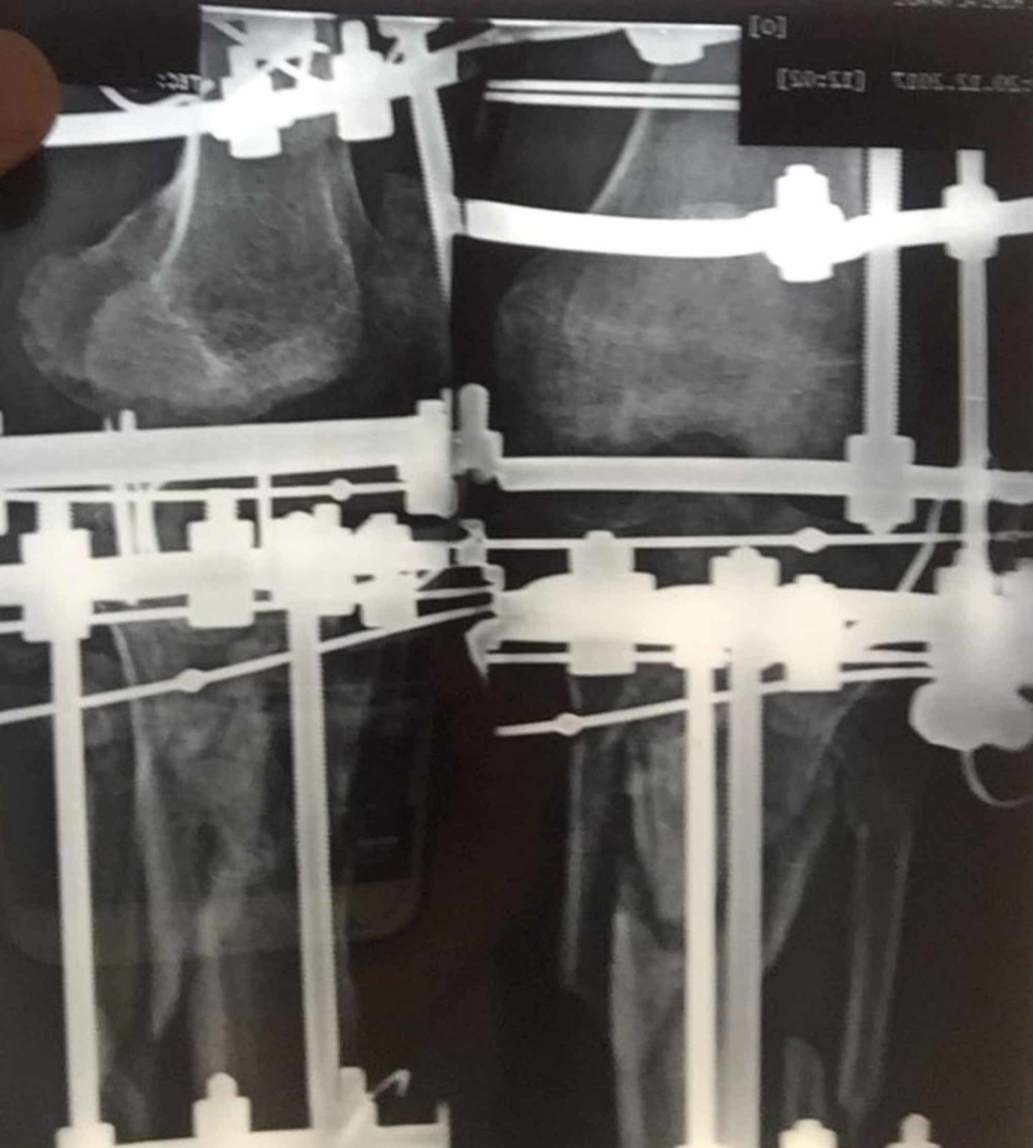
Status postoperative Ilizarov fixator with uni-focal distraction osteogenesis by ligamentotaxis

Axial deviation (varus deformity) was observed in one patient early (one week postoperatively), and this was corrected by the modification of the frame assembly under general anesthesia (Figure 3). One patient united at a 15° varus alignment.

## Discussion

The key to successful outcomes for bicondylar tibial plateau fractures are to restore the articular cartilage, preserve the biology, and obtain a painless, mobile, and aligned knee joint [10].

Open reduction and internal fixation were considered to be the best mechanical method of stabilization for bicondylar tibial plateau fractures. It has the advantage of an accurate reduction and stable fixation. However, it carries the risk of further soft-tissue damage and infection [11-13].

Not all bicondylar tibial plateau fractures will reduce with ligamentotaxis alone, and a limited open reduction with minimal periosteal stripping is sometimes necessary [14]. In our study, limited open reduction through a 5-6-cm incision was needed in nine cases, whereas bone grafting to support the elevated articular surface was needed in three (15%) cases. This is comparable to the study of Morandi and Pearse who reported elevation and bone grafting in 26% of cases in a series of 50 complex plateau fractures treated with Ilizarov fixation [15]. Several authors have identified factors that maximize the chances of a favorable outcome [16]. These factors include the amount of damage to soft tissues and articular cartilage, the accuracy of reduction, the stability of the knee joint, the stability of fixation, and the overall alignment of the limb [17]. All of these factors should be optimized in the care of tibial plateau fractures. Most of the reports include only low-energy or very few high-energy fractures. There is little reported information that focuses on the results of the treatment of high-energy fractures [18].

The magnitude of soft-tissue injury was also an important predictor of functional outcome. The technique for bicondylar fractures was originally performed through a single anterior incision, with subperiosteal dissection of the proximal tibia on both the medial and lateral sides. This massive soft-tissue stripping leads to the devascularization of bone and a high rate of infection. Infection rates markedly decreased and outcomes greatly improved when a less invasive technique was used; however, the risk of deep infection and soft-tissue complications is still present [19]. Lee et al. [20] reported on 36 tibial plateau fractures treated with a less invasive stabilization system. Two of them had a deep infection and one had extended skin necrosis and required plastic surgery. Most of the authors who had good results with the internal fixation of such fractures have used the external fixator as a preliminary step to help soft-tissue healing before internal fixation [21]. We prefer to use the external fixator as a definitive line of treatment and to avoid exposure to another surgery. Fixation of the fracture using the Ilizarov circular fixator avoids extensive soft-tissue dissection.

In a series by Dendrinos, 24 patients were treated with the Ilizarov circular fixator, and there was no incidence of osteomyelitis or septic arthritis. Chin et al. [17] reported similar results in 18 patients, none of whom developed wound dehiscence, infection, osteomyelitis, or septic arthritis. The current series is comparable to these studies in that no cases of wound dehiscence, infection, osteomyelitis, or septic arthritis were encountered.

Recent biomechanical studies proved that the fine-wire fixator provides adequate mechanical stability for the fixation of bicondylar tibial plateau fractures. The ring fixator provides good purchase in soft cancellous bone. The tensioned wires function as a scaffold in buttressing the subchondral bone, restore the intrinsic stability of the fracture site with a bridge device, and allow the patient to transfer his or her body weight through the scaffold to allow early weight-bearing.

The timing of surgery is one of the factors affecting the final outcome. This factor is one of the factors that can be controlled. In this study, we operated on our cases as early as possible to avoid the development of edema and to allow early soft-tissue recovery. Zura et al. [19] and Hak et al. [18], however, believed that early surgical reduction and firm fixation could retard further injury to local soft tissues. We deem that the majority of the soft tissues around the fractured tibial plateau have a relatively mild swelling within 12 h after injury. Therefore, internal fixation is considered eligible at this period, provided that local skin tension is not too high.

Varus deformity was observed during follow-up in one of the cases. A circular external fixator offers the unique possibility of correcting acutely or gradually any residual axial deviation at any plane, even torsional deformities.

## Conclusions

The decreased incidence of soft-tissue complications, early range of motion, early weight-bearing, and good functional recovery all compare favorably with other reported results and substantiate the recommendation that external fixation should be the treatment of choice for such injuries. A possible further advantage of this treatment modality is the fact that minimal dissection is performed and minimal metalware is left in situ.
